# Coronavirus disease 2019 in Italy: The Veneto model

**DOI:** 10.1017/ice.2020.225

**Published:** 2020-05-11

**Authors:** Ugo Grossi, Giacomo Zanus, Carla Felice

**Affiliations:** 1 Regional Hospital Treviso, University of Padua, Italy; 2 Centre for Neuroscience, Surgery and Trauma, Barts and the London School of Medicine & Dentistry, Queen Mary University of London, London, United Kingdom

*To the Editor—*In a Viewpoint article in the *Journal of the American Medical Association (JAMA)* regarding the third novel coronavirus (SARS-CoV2) outbreak in Wuhan, China, in December 2019, Drs Wu and McGoogan presented the key findings in a large case series of 72,314 infected individuals, refining the estimates in China to severe disease in 14% of cases and a case-fatality rate of 2.3%.^1^ Among the total case records, only 889 (1%) were classified as asymptomatic (ie, lacking typical symptoms including fever, dry cough, and fatigue), >5 times lower than those observed in Italy.^2^ After February 21, 2020, Italy became the hardest-hit country, with the highest death toll of 17,127 (>5 times as much as China) and 135,586 confirmed cases on April 7, 2020.

**Fig. 1.**
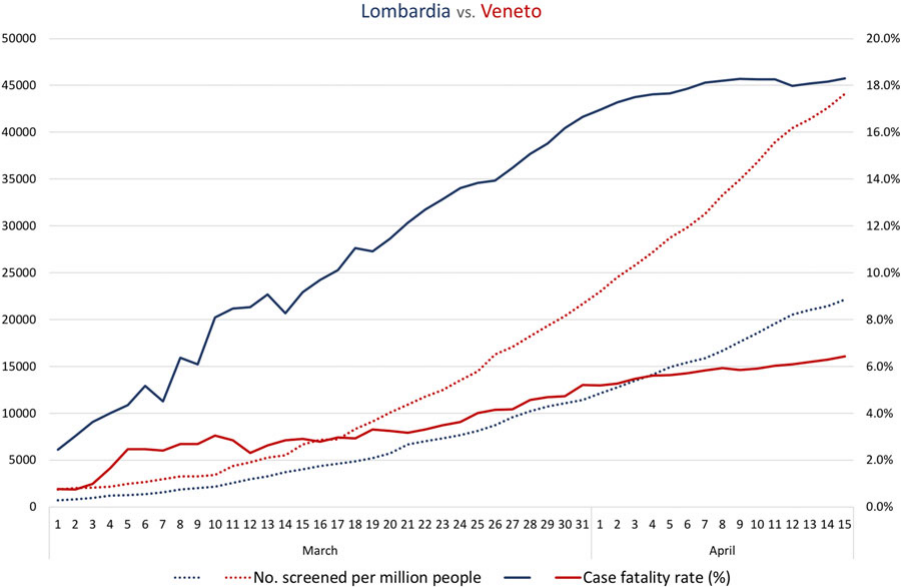
Timeline of number of tests for SARS-COV-2 per million people (dotted lines) and case fatality rate (solid lines) in Lombardia (blue) and Veneto (red). Source: Italian Civil Protection and Ministry of Health.

Recent evidence shows that 86% of all infections were undocumented prior to the Wuhan shutdown on January 23, 2020, representing the infection source for 79% of documented cases.^3^ It has now become evident that the percentage of mild or asymptomatic cases in developed societies, such as China and Italy, prior to major restrictions or control, was much higher than previously recognized. Although challenging without strict containment measures, identification of these subjects may slow the spread of SARS-CoV2. The cases of Veneto and Lombardia, contemporaneously affected neighboring regions in Italy, provide 2 telling examples with strikingly different endings. As of April 15, 2020, a total of 44,107 tests per million people have been performed in Veneto, which is double the number of tests conducted in Lombardia (Fig. 1). Indeed, in contrast to the neighboring region, Veneto adopted a large-scale population screening model at the beginning of the outbreak, allowing home isolation for a larger number of mild (or asymptomatic) cases (85% vs 60% of active cases according to the last estimates).^4^ This strategy may have avoided overwhelming the health system, with a consequent positive impact on the case fatality rate in Veneto, which is currently almost 3 times lower than that in Lombardia.

In fact, in line with previous modeling predictions,^5^ Lombardia did run out of intensive care beds at the end of March, resulting in the need to transfer critical patients to other Italian regions or European countries. Furthermore, between one-half and one-third of the daily national deaths are still being recorded in this region at this time.

At a time when Italy is facing its biggest challenge since World War II, the ‘Veneto model’ indicates that early mass screening for SARS-CoV-2 can make a positive difference, and it should be recommended to other countries responding to the COVID-19 pandemic.
